# A training for health care workers to integrate hepatitis B care and treatment into routine HIV care in a high HBV burden, poorly resourced region of Uganda: the ‘2for1’ project

**DOI:** 10.1186/s12909-022-03329-3

**Published:** 2022-04-20

**Authors:** Joan Nankya-Mutyoba, David Ejalu, Claude Wandera, Rachel Beyagira, Jacinto Amandua, Emmanuel Seremba, Kaggwa Mugagga, Andrew Kambugu, Alex Muganzi, Philippa Easterbrook, Ponsiano Ocama

**Affiliations:** 1 Department of Epidemiology & Biostatistics, School of Public Health, Makerere University College of Health Sciences, P.O. Box 7072, Kampala, Uganda; 2 Infectious Diseases Institute, Makerere University College of Health Sciences, Kampala, Uganda; 3 Program On Viral Hepatitis, Ministry of Health, Kampala, Uganda; 4 Department of Medicine, School of Medicine, Makerere University College of Health Sciences, Kampala, Uganda; 5 Global Hepatitis Program, WHO, Geneva, Switzerland

**Keywords:** Hepatitis B, Knowledge, Health care workers, Uganda

## Abstract

**Introduction:**

The “2for1” project is a demonstration project to examine the feasibility and effectiveness of HBV care integrated into an HIV clinic and service. An initial phase in implementation of this project was the development of a specific training program. Our objective was to describe key features of this integrated training curriculum and evaluation of its impact in the initial cohort of health care workers (HCWs).

**Methods:**

A training curriculum was designed by experts through literature review and expert opinion. Key distinctive features of this training program (compared to standard HBV training provided in the Government program) were; (i) Comparison of commonalities between HIV and HBV (ii) Available clinic- and community-level infrastructure, and the need to strengthen HBV care through integration (iii) Planning and coordination of sustained service integration. The training was aided by a power-point guided presentation, question and answer session and discussion, facilitated by physicians and hepatologists with expertise in viral hepatitis. Assessment approach used a self-administered questionnaire among a cohort of HCWs from 2 health facilities to answer questions on demographic information, knowledge and attitudes related to HBV and its prevention, before and after the training. Knowledge scores were generated and compared using paired t- tests.

**Results:**

A training curriculum was developed and delivered to a cohort of 44 HCWs including medical and nursing staff from the two project sites. Of the 44 participants, 20 (45.5%) were male, average age (SD) was 34.3 (8.3) with an age range of 22–58 years. More than half (24, 54.5%) had been in service for fewer than 5 years. Mean correct knowledge scores increased across three knowledge domains (HBV epidemiology and transmission, natural history and treatment) post-intervention. However, knowledge related to diagnosis and prevention of HBV did not change.

**Conclusion:**

A structured HBV education intervention conducted as part of an HIV/HBV care integration training for health care workers yielded improved knowledge on HBV and identified aspects that require further training. This approach may be replicated in other settings, as a public health strategy to heighten HBV elimination efforts.

**Supplementary Information:**

The online version contains supplementary material available at 10.1186/s12909-022-03329-3.

## Introduction

Infection with hepatitis B virus (HBV) is an on-going global public health challenge. Of the 257 million individuals that are infected worldwide, 60 million reside in Africa [1–3]. With chronic HBV, comes complications that significantly contribute to mortality, including cirrhosis, liver failure and liver cancer. Hepatitis B is responsible for the majority of liver cancers in the African region [4, 5], and these not only occur at a much younger age but are also associated with a poor prognosis [6]. This evidence implies that without strengthening HBV elimination efforts in this region, it will be difficult to avert future deaths from liver cancer in young African populations. The World Health Organization (WHO) global health sector strategy [7] aimed at eliminating the public health threat due to HBV by 2030 has recommended adopting a public health response to the disease. Emerging from this global strategy, HBV elimination targets for sub-Saharan Africa (SSA) revolve around preventing incident infections and providing appropriate care for those with prevalent infections [8, 9]. Several challenges in the path to achieving the 2030 global targets have been reported. Within the SSA context, these include low population awareness about HBV, inadequate HBV knowledge among health care personnel, and complexities around provision of timely HBV vaccination to newborns and lack of simplified service delivery models for HBV care and treatment. In order to achieve the 2030 HBV elimination goals, significant steps must be taken to close existing gaps and ensure progress. There is need to broaden coverage of HBV care services, and to boost the capacity of health care facilities and health care workers (HCWs) to detect and treat chronic HBV [10].

One strategy to promote access to HBV care and treatment is to build on and integrate with the HIV care infrastructure, which is relatively well-established across SSA. Health care workers need to be able to provide comprehensive HBV care across the continuum including on-going monitoring of those with chronic infection for signs of liver disease. Studies have shown that with adequate training, available HCWs could be optimally leveraged to provide continuous HBV care [11]. Training of HCWs could also help offset other socio-cultural dimensions within affected communities which continue to deter progress towards HBV elimination, such as patients’ low knowledge and awareness about HBV [12, 13], and HBV-related stigma [14]. When HCWs are well trained, they are confident to share this knowledge within their communities, and to relay accurate and professional advice to the general public and to those who need to access the services [15].

However, existing evidence shows that HCWs themselves have significant gaps in HBV knowledge. Studies from SSA have mostly found low HBV knowledge among HCWs [16, 17, 18] in addition to negative attitudes [19]. An Ethiopian study, however, showed high knowledge and positive attitudes towards HBV [20] These reports indicate, in the first place, that the specific knowledge gaps differ with each country, and therefore assessment of country-specific and where needed, sub-national HBV knowledge is relevant to gauge level of understanding, to identify training needs for various categories of HCWs and to enable evaluation of HBV education activities [21]. Secondly, these data suggest that poor knowledge may influence stigma and negative attitudes towards HBV among HCWs. Several studies have assessed HBV-related knowledge and recommended education interventions to address identified information deficits. Fewer studies have, nonetheless, examined whether HBV education interventions result in knowledge improvement among trainees. The majority of these studies have been done among at-risk minority Asian populations in the USA [22–28]. In Africa, studies that report on public health interventions for HBV prevention are generally uncommon. A few published studies were done in Egypt [29–32], and one recent study in Jos region of Nigeria [33]. Of these studies, only one evaluated the intervention among HCWs [32]. This therefore necessitates an evaluation of knowledge uptake among HCWs in specific settings in order to inform programs for HBV education.

Within a wider pilot program for integrating HBV care into HIV programs in public health facilities in Uganda, we designed a HBV education curriculum and trained HCWs in West Nile region of Uganda. The training component consisted of an initial education workshop for HCWs involved directly or indirectly in the care of HIV- and HBV-infected patients, followed by continued medical education sessions as part of on-going support for integration process. Training modules aimed to emphasize both rationale and areas for integration of care. As part of the training we performed a pre- and post-test HBV knowledge evaluation in the initial cohort of HCWs. The objective of this research was to describe the HBV/HIV education curriculum content and to assess its effect on HBV knowledge among the HCWs.

## Methods

### Study setting, design and population

#### Study setting: The “2for1” project

This demonstration project, named “2for1” aimed to impact two diseases, that is, HIV and hepatitis B through delivery of a simplified integrated care model in one setting. The “2for1” project was designed as a demonstration project as part of a collaboration between the Uganda Ministry of Health, WHO and researchers from the College of Health Sciences and the Infectious Diseases Institute, Makerere University. It is a pilot project to examine the feasibility and effectiveness of integrating care and treatment of HBV into routine HIV care services. It is based on (i) the scientific knowledge that these two infections are similar in terms of routes of transmission, long-term treatment and common medications; (ii) the lack of adequate logistics and health care work force for effective treatment, monitoring and follow-up of HBV infected individuals. The integration into one care system, it was proposed, might improve care for both infections and afford more efficient use of available resources. It was purposely elected to be performed in West Nile region of Uganda because of the high prevalence of HBV in the region, with up to 19% of the population found to be having chronic hepatitis B in 2004 [34]. The Government of Uganda performed mass HBV screening of the population in the region for all persons aged 14 years and above with intention of vaccinating all those who did not demonstrate infection (negative for hepatitis B surface antigen -HBsAg) [35]. A significant proportion of those identified as infected both in Arua and Koboko districts were lost to follow up due to limited services for HBV despite availability of dedicated HBV clinics in the same hospitals. Both Arua Regional referral hospital (RRH) and Koboko District hospital (DH) have a functional HIV care structure, supported by PEPFAR-funded partners. The project focused on engaging HCWs, preparing them for HIV/HBV integrated care and management through specific training and support through the integration process.

The study was conducted in two public health care facilities, Arua RRH and Koboko DH, a district hospital, both located in West Nile region, approximately 300 miles northwest of Kampala. West Nile region shares boundaries with the Democratic Republic of Congo (DRC) and South Sudan. It has a population of 2,988,300 people and is one of the highest HBV prevalent regions in Uganda, with a reported prevalence higher than the national average. Arua RRH is a large health facility [36] that provides both primary and specialized care for patients within the district and some neighboring districts, while Koboko DH is a district hospital that serves a border population intersecting Uganda, South Sudan and the DRC.

### Study design, population and participant recruitment

This training intervention was designed as a non-randomized, pre- and post- education intervention delivered as part of the demonstration project to study the feasibility of integrating HBV care into routine HIV care system.

Our study population included personnel from various hospital departments that are directly or actively involved in the provision of services to patients with HBV or HIV infection at these health facilities. Out of the total of 51 HCWs, we randomly selected seven HCWs including four in Arua RRH which is the larger facility, and three in Koboko DH to remain at the facilities for continuity of service delivery on the day of training. The remaining 44 HCWs were all invited to participate in the training and they all attended. They included Physicians, Medical Officers, Nursing and Clinical Officers, Laboratory technicians, Enrolled nurses/midwives, pharmacy technicians and records/data officers. The staff who serve as data officers are trained nurses who are also actively involved in providing care services including patient counseling and education as part of task-sharing, in addition to their main duty of data entry and management of patient clinical records. We therefore included them in the training.

With authorization from the top management of these facilities, eligible service providers were identified and letters of invitation were written and sent out to them for a one-day training through their supervisors. All the invited cadres turned up for the training.

### Training curriculum design and instructional approach

An initial phase in implementation of this project was the development of a specific training program. We developed a specific training curriculum, guided by literature review and experts in clinical and academic training in the area of Hepatology. The curriculum aimed to improve HBV knowledge, to raise understanding of commonalities between HIV and HBV and the importance of merging care for both chronic infections. It had four modules, namely HBV natural history and disease transmission, laboratory diagnosis, care and treatment as well as prevention. The “disease natural history” module emphasized [1] that both HBV and HIV are transmitted in similar ways, [2] and it discussed the influence of HIV on HBV and vice versa in the natural history of both infections. The “laboratory diagnosis” module highlighted the similar platforms for laboratory diagnosis of HIV and HBV using ELISA, the HIV viral load and HBV viral load, the use of similar transportation systems (the hub system) for viral load samples and the same processing facility for both viral load tests, the central public health laboratories. This single system for sample transportation and analysis would ease operations for care integration. The "care, treatment and prevention" modules discussed commonalities in relation to continuity of care, antiviral medications, process of obtaining drugs and a patient education and counseling message with components of prevention of both infections.

### Pre-intervention

Following provision of informed consent, all participants were provided with a self-administered questionnaire (SAQ) and requested to complete it, independently. The SAQ was developed by experts including academic hepatologists, researchers and clinicians and was based on standard documents including WHO guidelines [37, 38] and guidelines developed by the Uganda Ministry of Health. The SAQ had 6 sections, covering background information (age, sex, health facility, Health care provider category, duty station and years in service). The second section was dedicated to the “HBV disease epidemiology and transmission” and “natural history” and each had 9 questions with “Yes”/ “No”/ “Don’t know” responses. The rest of the sections (5, 8 and 6) focused on HBV diagnosis, treatment and prevention respectively with responses of questions with “Yes”/ “No”/ “Don’t know” responses provided at the end of each question. The tool also had two questions that assessed health care workers’ attitudes towards HBV-infected persons. The tool was piloted on a group of HCWs outside the study region, and was revised to improve question item wording, flow and clarity. The tool has been provided as supplementary material.

### HBV education intervention

This intervention was a one-day HBV-focused training delivered by expert Physicians and academic hepatologists using the developed curriculum. The training was provided as a didactic seminar, guided by a power-point presentation, coupled with illustration on flip charts, and participants were provided with note books to make personal notes. The presentation was followed by a question and answer session in which participants made inquiry on aspects of HBV they did not understand well, and an interactive discussion session which allowed free learning to take place. The training had breaks of 30 min every after 2 h and participants were also provided with the information in print format.

### Post-intervention

**Fig. 1:**
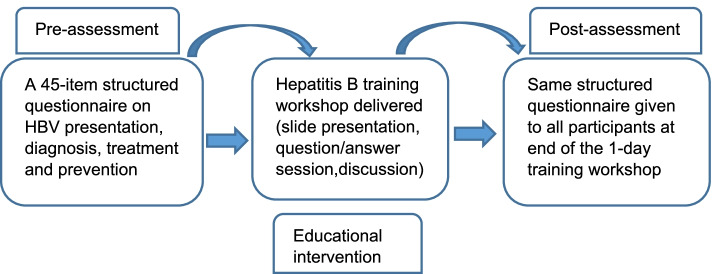
Schema showing pre-training HBV knowledge assessment, education intervention and post-training assessment of health care workers in West Nile region, Uganda

Following the training, participants were re-convened and provided with the same SAQ they had received prior to the training, and asked to complete it for a second time. All health care workers who participated in the training completed the same questionnaire after the training. Figure 1 shows the processes participants were taken through.

### Data analysis

Data were cleaned and analysed using SPSS. Descriptive analysis was done, and frequencies generated with mean and standard deviation (SD) for continuous variables and proportions for categorical variables. Scores were generated by assigning a score of one to all “Yes” responses (correct response), and a score of zero to “No” or “Don’t know” responses for question items assessing knowledge. McNemmar’s paired t-test was used to compute differences in mean scores of correct responses for each knowledge component at pre- and post- intervention, with 95% confidence intervals. Regarding analysis of attitudes, responses that were “Yes” or “Don’t know” to each of the attitudes questions were classified as “negative” and scored one, meaning presence of stigmatizing attitudes, while responses of “No” were classified as “positive” and given a score of zero, meaning absence of stigmatizing attitudes. Proportion of respondents with “negative” attitude was estimated. To analyse differences in knowledge gain by age, we classified HCWs into those who were younger (aged below 30 years of age) and those who were older (aged at least 30 years old). Analysis of knowledge gain by duration in service, we classified HCWs into two broad categories, comparing those who were relatively new in service (served for up to 5 years) to those who had longer duration in service (more than 5 years).

### Study approval

Both the study protocol and all tools received ethical review and clearance from the School of medicine research and ethics committee (approval number REC 2018–185), the Scientific Review Committee of the Infectious Diseases Institute and the Uganda National Council for Science and Technology (approval Ref SS 4986).

## Results

A total of 44 out of 51 HCWs who serve the HIV and HBV clinics were invited to participate in the training (participation proportion of 86%). All participants completed both the pre- and post-intervention questionnaire, providing 100% response fraction. Of these, 20 (45.5%) were male (Table 1). The average age (standard deviation) was 34.3 (8.3) years. Slightly more than half (24, 54.5%) of respondents had been in service for fewer than 5 years. The rest of the baseline characteristics are shown in Table 1.

**Table 1:** Characteristics of Health Care Workers who participated in the HBV education training in Arua, West-Nile region

Characteristic	Mean	SD
**Age (years)**	34.3	8.3
	**N**	**%**
**Sex**		
Male	20	45.5
Female	24	54.5
**Age-group**		
20–29	17	38.6
30–39	14	31.8
40–49	10	22.7
50–59	3	6.8
**Health care worker category**		
Physician/medical officer	6	13.6
Clinical officer	9	20.5
Midwife	4	9.1
Nurse	8	18.2
Lab-technician	4	9.1
Pharmacy technician	4	9.1
Data/records officer	3	6.8
Patient counselors	4	9.1
Community outreach worker	2	4.5
**Duration in service (years)**		
< 5	24	54.5
5–10	11	25.0
> 10	9	20.5
**Health facility**		
Arua	28	64.0
Koboko	16	36.0

### “Training for integration” curriculum content

The specific “Training for Integration” curriculum content is summarized in Table 2. It consisted of 5 modules covering HBV disease epidemiology and natural history, diagnosis, care and treatment, prevention, and cross-cutting issues relating to integration. For modules 1 to 4, training aimed at improving overall knowledge by highlighting commonalities between HIV and HBV in aspects of disease natural history and transmission, diagnosis, treatment and prevention.

**Table 2:** Content of hepatitis B “Training for integration” Curriculum for Health Care Workers in West Nile Region, Uganda

Module	Module content
**Module 1** Natural history, epidemiology and transmission of infection	I. Emphasized burden of HIV, of HBV, of dual infection, and similarities between HBV and HIV infection transmission II. The influence of HIV on HBV natural history, and the influence of HBV on HIV natural history
**Module 2** Laboratory diagnosis	I. Basics of laboratory diagnosis for HBV II. Similar platforms for diagnosis of HIV and HBV (ELISA), III. HIV viral load and HBV viral load IV. Similar transportation systems for samples (the hub system) for viral load samples V. Same processing facility for viral load tests (Central Public Health Laboratories)
**Module 3** Care and treatment	I. Covered for HBV and HBV-HIV co-infection II. Both being chronic diseases with no cure but life-long care and treatment III. The HIV “test and treat” strategy, versus the HBV treatment strategy that requires additional tests to decide who is eligible for treatment IV. Medications for HBV treatment provided in public hospitals by the Government and ART by both Government and Partners
**Module 4** Prevention	I. Commonalities between prevention of HIV and of HBV, including preventing mother-to-child HBV transmission II. Community-linkage for continued follow-up and prevention of complications for HBV mono-infected persons III. Community engagement Community networks of HBV (treatment peer support, community groups to have patients’ voice) to be woven into existing structure for HIV Need for continuous monitoring emphasized, and to remain in care Need for raising community awareness, to eliminate erroneous disease perceptions and fight stigma addressed as part of curriculum, through local radio-based programs, where individuals calls-in and have their questions and concerns discussed as part of community engagement
**Module 5** Cross-cutting issues, rationale, planning and coordination of integration	I. Why integrate? (i)Significant overlap between both infections and the need to offer care for both infections in a well-coordinated care system. (ii) Need for a public health response, to give the widest possible reach for HBV care, treatment and continuous monitoring would be efficiently met through care integration II. HIV as a “high profile” disease (Well-structured clinics all over the country; funded laboratories and pharmacy, strong patient and community networks, clinician task-shifting and data capture services) versus HBV as a “neglected” disease (Runs a silent course, with high levels of stigma and discrimination, lacks all infrastructure, apart from free tenofovir, has insufficient media representation and advocacy among health workers, patients and at political level) III. Leverage on the well-developed infrastructure for HIV to offer HBV services to all who need it IV. Co-planning, training and coordination for integrated HIV and HBV service delivery across the care continuum

Module 4, on disease prevention emphasized the role of community engagement to raise awareness on both infections and the benefits of offering integrated care to the same community. Module 5 had content that explained the rationale and capacity of the existing HIV care system to respond to both infections. It showcased HBV as a neglected infection that could benefit from being appended onto the already existing HIV care infrastructure, for a maximized response. It also discussed gaps in Health worker training, coordination and planning for sustained integrated care both at the clinic level and the community level.

### HBV-related knowledge

#### Total knowledge

Overall, the mean (SD) total knowledge score was 27.2 (5.3) out of 35 prior to receiving training, and it rose to 30.6 (3.8) after receiving training. There were statistically significant increase in mean scores of total knowledge across subcategories of age, gender, duration in service, HCW cadre and health facility (Table 3).

**Table 3:** Pre- and post- intervention mean scores of participants’ total knowledge by baseline characteristics

Characteristic	Pre-test mean score (standard deviation)	Post-test mean score (standard deviation)	*p*-value
**Gender**			
Male	29.3 (5.4)	31.8 (4.0)	**0.011**
Female	25.5 (4.7)	29.6 (3.4)	** < 0.001**
**Age (years)**			
≤ 30	26.6 (5.3)	29.9 (3.6)	** < 0.001**
> 30	27.9 (5.3)	31.2 (4.0)	**0.001**
**Duration in service (years)**			
≤ 5	27.9 (5.7)	31.2 (4.4)	** < 0.001**
> 5	26.6 (5.0)	29.9 (2.9)	**0.005**
**Health facility**			
Arua	26.4 (5.2)	30.5 (3.7)	**0.000**
Koboko	28.7(5.4)	30.7(4.1)	**0.013**

#### Domain-specific knowledge

Analysis of mean knowledge scores by knowledge domain is shown in Table 4. There was a statistically significant increase in mean scores of three knowledge domains post-intervention; epidemiology and transmission of HBV (from 8.5 (1.7) to 9.5 (0.8) out of 10 scores, *p* < 0.001), natural history of HBV (from 4.0 (1.8) to 4.7 (1.6) out of 8 scores, *p* = 0.011) and treatment of HBV (from 5.5 (1.5) to 7.0 (1.2) out of 8 scores, *p* < 0.001). However, knowledge related to diagnosis and prevention of HBV did not change.

**Table 4:** Pre- and post-intervention mean scores of HBV knowledge domains

Knowledge domain	Pre-test mean score (standard deviation)	Post-test mean score (standard deviation)	*p*-value
Epidemiology and transmission	8.5(1.7)	9.5 (0.8)	** < 0.001**
Natural history of HBV	4.0(1.8)	4.7 (1.6)	**0.011**
Diagnosis of HBV	2.4(1.1)	2.5 (1.2)	0.685
Treatment of HBV	5.5(1.5)	7.0 (1.2)	** < 0.001**
Prevention of HBV	4.2(1.1)	4.0 (1.0)	0.384

Our analysis of sub-categories of age, gender, years in service and health facility, showed that there was an increase in knowledge of HBV epidemiology and transmission, and of HBV treatment even across categories of age, gender, duration in service and health facility (Table 5). Regarding natural history of HBV, there was an increase in mean knowledge scores (from 3.6 (1.9) to 4.3(1.7) out of 9, *p* = 0.045) among female HCWs, but not among male HCWs. There was increase in knowledge among those older than 30 years, but not among those aged 30 years and younger. Among health facilities, there was a significant increase in mean knowledge scores among HCWs in Arua, but not Koboko hospital. Regarding knowledge of HBV diagnosis, there was no gain in knowledge across any category. Knowledge of HBV prevention equally did not change, and it significantly decreased after the training, among HCWs of Koboko hospital, (from 4.6 (0.9) to 3.9 (1.1) out of 5, *p* = 0.022). Mean scores of HBV prevention generally decreased among HCWs across categories of age, gender and years in service.

**Table 5:** Pre- and post-test mean scores of participants’ domain-specific knowledge, by sex, age and years in service

			**Pre-test score**	**Post-test score**	
**Variable**	**Category**	**Frequency** **n (%)**	**Mean (Standard deviation)**	**Mean (Standard deviation)**	***p*- value**
**Epidemiology and Transmission**					
**Gender**	Male	20 (45.5%)	9.0 (1.5)	9.4(0.9)	**0.05**
	Female	24 (54.5%)	8.2 (1.8)	9.5 (0.7)	**0.001**
**Age (years)**	≤ 30	22 (50.0%)	8.5 (1.5)	9.3 (0.6)	**0.008**
	> 30	22 (50.0%)	8.5 (1.9)	9.5 (0.9)	**0.007**
**Duration in service (years)**	≤ 5	24 (54.5%)	8.8 (1.5)	9.5 (0.8)	**0.008**
	> 5	20 (45.5%)	8.2 (1.8)	9.4 (0.8)	**0.006**
**Health facility**	Arua	28 (64.0%)	8.6 (1.5)	9.4 (0.8)	**0.016**
	Koboko	16 (36.0%)	8.4 (1.8)	9.6 (0.8)	**0.002**
**Natural history of HBV**					
**Gender**	Male	20 (45.5%)	4.5 (1.5)	5.2 (1.5)	0.131
	Female	24 (54.5%)	**3.6 (1.9)**	**4.3 (1.7)**	**0.046**
**Age (years)**	≤ 30	22 (50.0%)	4.0 (1.6)	4.2 (1.4)	0.535
	> 30	22 (50.0%)	**4.0 (1.9)**	**5.2 (1.7)**	**0.004**
**Duration in service (years)**	≤ 5	24 (54.5%)	**4.0 (1.6)**	**4.7 (1.7)**	**0.044**
	> 5	20 (45.5%)	4.1 (1.9)	4.7 (1.6)	0.124
**Health facility**	Arua	28 (64.0%)	**3.8 (1.9)**	**4.7 (1.8)**	**0.008**
	Koboko	16 (36.0%)	4.4 (1.4)	4.6 (1.5)	0.628
**Diagnosis of HBV**					
**Gender**	Male	20 (45.5%)	2.9 (1.3)	3.0 (1.2)	0.649
	Female	24 (54.5%)	2.0 (0.9)	2.1 (1.1)	0.869
**Age (years)**	≤ 30	22 (50.0%)	2.1 (1.0)	2.7 (1.2)	0.589
	> 30	22 (50.0%)	2.7 (1.2)	2.7 (1.3)	1.000
**Duration in service (years)**	≤ 5	24 (54.5%)	2.5 (1.1)	2.7 (1.3)	0.462
	> 5	20 (45.5%)	2.4 (1.1)	2.3 (1.2)	0.847
**Health facility**	Arua	28 (64.0%)	2.3 (1.1)	2.5 (1.2)	0.312
	Koboko	16 (36.0%)	2.4 (1.2)	2.6 (1.3)	0.509
**Treatment of HBV**					
**Gender**	Male	20 (45.5%)	6.1 (1.4)	7.2 (1.0)	**0.001**
	Female	24 (54.5%)	4.9 (1.4)	6.8 (1.2)	** < 0.001**
**Age (years)**	≤ 30	22 (50.0%)	5.3 (1.8)	6.9 (1.2)	** < 0.001**
	> 30	22 (50.0%)	5.6 (1.3)	7.1 (1.1)	** < 0.001**
**Duration in service (years)**	≤ 5	24 (54.5%)	5.7 (1.5)	6.9 (1.3)	**0.001**
	> 5	20 (45.5%)	5.1 (1.6)	7.1 (0.9)	** < 0.001**
**Health facility**	Arua	28 (64.0%)	5.2 (1.5)	7.1 (1.1)	** < 0.001**
	Koboko	16 (36.0%)	7.1 (1.1)	6.8 (1.2)	** < 0.001**
**Prevention of HBV**					
**Gender**	Male	20 (45.5%)	4.3 (1.2)	4.3 (0.9)	0.825
	Female	24 (54.5%)	4.2 (1.0)	3.9 (1.0)	0.397
**Age (years)**	≤ 30	22 (50.0%)	4.0 (1.0)	4.1 (1.0)	0.880
	> 30	22 (50.0%)	4.4 (1.1)	4.0 (0.8)	0.088
**Duration in service (years)**	≤ 5	24 (54.5%)	4.3 (1.0)	4.1 (0.9)	0.632
	> 5	20 (45.5%)	4.2 (1.1)	4.0 (0.8)	0.447
**Health facility**	Arua	28 (64.0%)	4.0 (1.1)	4.1 (0.8)	0.526
	Koboko	16 (36.0%)	**4.6 (0.9)**	**3.9 (1.1)**	**0.022**

### Attitude towards HBV-infected persons

Overall, only 2 out of 44 participants had negative attitudes towards HBV-infected persons prior to receiving the HBV training, and none of them had negative attitudes post-training. These cadres were data officers, one was male, and one female, and had been in service for less than 5 years.

## Discussion

In a region with high HBV prevalence and limited funds for HBV prevention, a structured HBV-focused training resulted in overall gain in HBV-related knowledge and a decline in stigmatizing attitudes among HCWs. We found statistically significant increase in mean scores of total knowledge across subgroups of gender, age and years in service. However, the differences in specific knowledge domains pre- and post-education intervention varied, with gains in knowledge of HBV epidemiology and disease transmission, disease natural history and treatment, while there was no knowledge gain in domains of HBV diagnosis and prevention. The WHO guidelines [7, 37, 38] were launched with the aim of supporting the initiation, augmentation and expansion of HBV prevention, care and treatment services by both country managers and front-line HCWs who care for HBV-infected patients. Based on these protocols, HCWs should be able to diagnose HBV, triage patients who need treatment and those who need regular monitoring, recommend the most suitable anti-viral therapies as well as counsel patients and their families on HBV preventive behaviours, avoiding stigma and providing on-going support to affected individuals. Our finding of overall improvement in HBV-related knowledge among HCWs is similar to what was observed among medical interns in Egypt [32] and in other settings [39]. However, we found, in this sample of HCWs, poor understanding of tests performed to detect HBV infection, interpretation of HBV serologic assays including HBV surface antigen, core antibody, HBV e antigen, and HBV viral load, both before and after receiving the HBV training. Similar to our study, Konlan et al. [40] showed that among 109 Nurses in Ghana, only 12.1% had knowledge of treatment following accidental exposure to HBV, and 87% of them had never received specific training on post-exposure treatment. Correct interpretation of HBV test results is important to HCWs, given their role in educating the public, counseling patients and making treatment decisions.

Low knowledge about HBV and its prevention has been reported among HCWs elsewhere [16, 41–44]. What has not been sufficiently documented particularly in SSA, is whether HCW-focused HBV education interventions improve knowledge and attitudes related to HBV and its prevention. Wang et al. [45] assessed a health care provider-focused online HBV training module and reported pre-existing knowledge deficits among Chinese physicians and nurses, similar to our study. This online training improved HCW knowledge. Sim et al. [46] used a cartoon and graphical illustration-type of online HBV training for HCWs in Australia and found that this intervention improved HBV knowledge among HCWs. Contrary to these two studies, we used a more traditional, face-to-face facilitator-led, instructional model mainly because this approach is more realistic in our rural settings, where HCWs have limited access to online resources. In a study by Taylor et al. [22], traditionally trained community health workers provided HBV education to their communities, resulting in knowledge improvement.

Our pre-training knowledge assessment exposed gaps in knowledge related to HBV diagnosis and prevention, and these gaps persisted post-education intervention, unlike in the Wang and Sim studies. In addition, knowledge elements related to the natural history of HBV were not uniformly understood, for instance knowledge improvement was realized among HCWs in Arua, but not in Koboko hospital, and among those older than 30 years of age, but not those aged 30 years or younger. A likely explanation for lower knowledge among HCWs in Koboko is that they may have had less previous exposure to HBV-related information, as this is a newly upgraded district hospital with less resources, fewer senior cadres and opportunities for accessing learning, unlike Arua hospital which is a well-established regional referral hospital with more senior cadres of HCWs.

In terms of stigmatizing attitudes, we found low levels of stigmatizing attitudes among HCWs. In addition, stigmatizing attitudes were observed among data officers, who are not front-line HCWs. Correlative to knowledge, we observed positive changes in attitudes among HCWs after the training. Unlike our findings, studies in India documented the existence of stigmatizing attitudes directed towards HBV-infected patients among physicians [47], and higher HBV knowledge to be associated with positive attitudes towards HBV infected patients [48]. Several hospital staff in Uganda’s health facilities are not front-line care givers, but intersect routinely with HBV-infected patients, and may participate in providing supportive care to them, as part of task-sharing, when health facilities are under-staffed. Trainings that aim to build their capacity and their patient communication skills should be considered, since they are part of the health system.

We note the following limitations in this study. First, the study design could not eliminate possibility of confounding, hence the findings may mostly serve to highlight areas for further inquiry through more rigorous methods. Second, we only assessed immediate changes in knowledge and attitudes, and coupled with our relatively small sample size, our findings do not sufficiently inform effect of training on long-term HBV-related attitudes and knowledge retention. The observed gains in HBV knowledge and declines in stigmatizing attitudes may, however, become more apparent with a larger sample size. Nonetheless, very few studies in SSA have assessed whether facility-based training improves HBV knowledge or reduces stigmatizing attitudes and behaviour of HCWs in the process of caring for HBV patients. This work will therefore inform policies and programs that aim to strengthen HCWs’ capacity for HBV prevention.

## Conclusion

A structured, facility-based HBV-focused education intervention conducted as part of HIV/HBV care integration training for HCWs in West Nile region of Uganda yielded improved knowledge about HBV and its prevention and more positive attitudes towards HBV-infected patients. It also identified specific areas where HCWs lacked knowledge, requiring focused training on domains that did not significantly change pre- and post- training to make the integration successful. This approach may be replicated in other settings, as a public health strategy to heighten HBV elimination efforts.

## Supplementary Information

**Additional file 1.**

## Data Availability

The data supporting the conclusions of this report are all included within this article and the supplementary material.
