# Supplementary material for: A training for health care workers to integrate hepatitis B care and treatment into routine HIV care in a high HBV burden, poorly resourced region of Uganda: the ‘2for1’ project
Source: BMC Med Educ. 2022 Apr 20;22:297. doi: 10.1186/s12909-022-03329-3 (PMC9020110; doi:10.1186/s12909-022-03329-3)
Supplement: Supplementary file 1 — Additional file 1. [file 12909_2022_3329_MOESM1_ESM.docx]

**Appendix 2. Questionnaire: Trainees**

**PROMOTING ACCESS AND SCALE UP OF HEPATITIS B CARE AND TREATMENT IN UGANDA: THE 2FOR1 PROJECT**

Participant number………………………..

Date……………………………………….

**A: Baseline information (Tick the appropriate number)**

1. District: District: Arua (1) Koboko: (2)
2. Gender: M (1) F (2)
3. Age……
4. Years of service…………
5. Health worker category : Physician (1) Medical officer (2) Clinical officer (3) Others (specify) (4) ……………………
6. Category of work station: Reginal Hospital (1) District Hospital (2) Health Centre IV (3) Health Centre III (4) Others (5) ………….. (Specify)

***B. Hepatitis B in Uganda: epidemiology and transmission***

1. Hepatitis B virus is found throughout the country: Yes (1) No (2) Don’t know (3)
2. In the world hepatitis B is found only in Africa: Yes (1) No (2) Don’t know (3)
3. The infection has just is new infection in Uganda: Yes (1) No (2) Don’t know (3)
4. In Uganda most infection with hepatitis B has been seen in Northern and Northeastern Uganda: Yes (1) No (2) Don’t know (3)
5. Hepatitis B spreads by sharing food, greetings, touching an infected person: Yes (1), No (2) Don’t know (3)
6. Hepatitis B and HIV share modes of transmission: Yes (1) No (2) Don’t know (3)
7. If a person dies of hepatitis B, the body needs to be handled in a similar way a person who dies of Ebola should be handled: Yes (1) No (2) Don’t know (3)
8. In Uganda currently blood for transfusion is not adequately tested for hepatitis B before transfusion: Yes (1) No (2) Don’t know (3)
9. Commonest mode of transmission in Africa is from an Infected mother to the baby: Yes (1) No (2) Don’t know (3)

**Attitude towards HBV patients**

1. Persons infected with hepatitis B should not be isolated on the basis of the fact that they are infected: Yes (1) No (2) Don’t know (3)
2. Teachers should send away pupils/students who are infected with hepatitis B from school because they will spread the infection to those not yet infected: Yes (1) No (2) Don’t know (3)

**C. Natural history**

1. Chronic Hepatitis B infection is defined by presence of cirrhosis: Yes (1) No (2) Don’t know (3)
2. Irrespective of the age of acquisition, all persons exposed to hepatitis B will have continue to have lifelong infection: Yes (1) No (2) Don’t know (3)
3. It is expected that when hepatitis B exposure takes place, persistence of Hepatitis B surface antigen beyond 6 months defines chronic hepatitis B infection: Yes (1) No (2) Don’t know (3)
4. When hepatitis B is acquired after 5 years of age, the infection is usually eliminated from the body through human immune system: Yes (1) No (2) Don’t know (3)
5. Hepatitis B is only one of the many causes of chronic liver diseases: Yes (1) No (2) Don’t know (3)
6. All persons with hepatitis B will end up with complications in their liver inclusive of cirrhosis, hepatocellular carcinoma and or liver failure : Yes (1) No (2) Don’t Know (3)
7. Close to 30% of person with chronic hepatitis B tend to be inactive carriers of hepatitis B: Yes (1) No (2) Don’t know (3)
8. HIV increases the progression of liver diseases to complications: Yes (1) No (2) Don’t know (3)
9. Persons with liver cancer induced hepatitis should be placed on antiviral therapy: Yes (1) No (2) Don’t know (3)

**D. Diagnosis of hepatitis B infection**

1. Hepatitis B core antibody is the initial test to define whether someone has hepatitis B or not: Yes (1) No (2) Don’t know (3)
2. A negative test for hepatitis B e antigen means infection has been eliminated: Yes (1) No (2) Don’t know (3)
3. The viral load for hepatitis B virus is required for all persons: Yes (1) No (2) Don’t know (3)
4. All HIV infected persons should be tested for hepatitis B and vice versa: Yes (1) No (2) Don’t know (3)
5. The viral load is the most important test to determine treatment initiation: Yes (1) No (2) Don’t know (3)

**E. Treatment of hepatitis B**

1. Treatment for hepatitis B is required in all persons who test positive for hepatitis B surface antigen: Yes (1) No (2) Don’t know (3)
2. If treatment is required it is taken for 6 months : Yes (1) No (2) Don’t know (3)
3. HIV infected patients who are co-infected with hepatitis B should be initiated on antiretroviral therapy irrespective of HIV and or hepatitis B viral load: Yes (1) No (2) Don’t know (3)
4. Hepatitis B can be cured with current available treatment options: Yes (1) No (2) Don’t know (3)
5. Current drugs for hepatitis B include tenofovir and entecavir : Yes (1) No (2) Doing know (3)
6. For hepatitis B mono-infected patients treatment should be in combination: Yes (1) No (2) Don’t know (3)
7. HIV and Hepatitis B co-infection requires use of at least 2 drugs with activity against both HIV and hepatitis B: Yes (1) No (2) Don’t know (3)
8. When witching treatment for HIV infection, drugs effective against hepatitis B should not be removed from the switched combination: Yes (1) No (2) Don’t know (3)

**F. Prevention**

1. Hepatitis B vaccine is the most effective method of preventing infection: Yes (1) No (2) Don’t know (3)
2. Hepatitis B vaccination is currently not available for children in Uganda: Yes (1) No (2) Don’t know (3)
3. All adults should be given hepatitis B vaccine: Yes (1) No (2) Don’t know (3)
4. Persons who are constantly receiving blood transfusion should be vaccinated against hepatitis B if they are not exposed: Yes (1) No (2) Don’t know (3)
5. Persons who get accidentally exposed to HBV should be given hepatitis B Immunoglobulin and also vaccinated against hepatitis B: Yes (1) No (2) Don’t know (3)
